# Supplementary material for: Transcriptomic Analysis Reveals Early Alterations Associated with Intrinsic Resistance to Targeted Therapy in Lung Adenocarcinoma Cell Lines
Source: Cancers (Basel). 2024 Jul 8;16(13):2490. doi: 10.3390/cancers16132490 (PMC11240825; doi:10.3390/cancers16132490)
Supplement: Supplementary file 1 [file cancers-16-02490-s001.zip › cancers-3050799-supplementary.docx]

**Supplementary Materials**

**Transcriptomic analysis reveals early alterations associated with intrinsic resistance to target therapy in lung adenocarcinoma cell lines**

Mario Perez-Medina**^1,2‡^**, Jose S. Lopez-Gonzalez**^1‡^**, Jesus J. Benito-Lopez**^1,3‡^**, Santiago Avila-Rios**^4^**, Maribel Soto-Nava**^4^**, Margarita Matias-Florentino**^4^**, Alfonso Mendez-Tenorio**^5^**, Miriam Galicia-Velasco**^1^**, Rodolfo Chavez-Dominguez**^1^**, Sergio E. Meza-Toledo**^2^**, and Dolores Aguilar-Cazares**^1^***


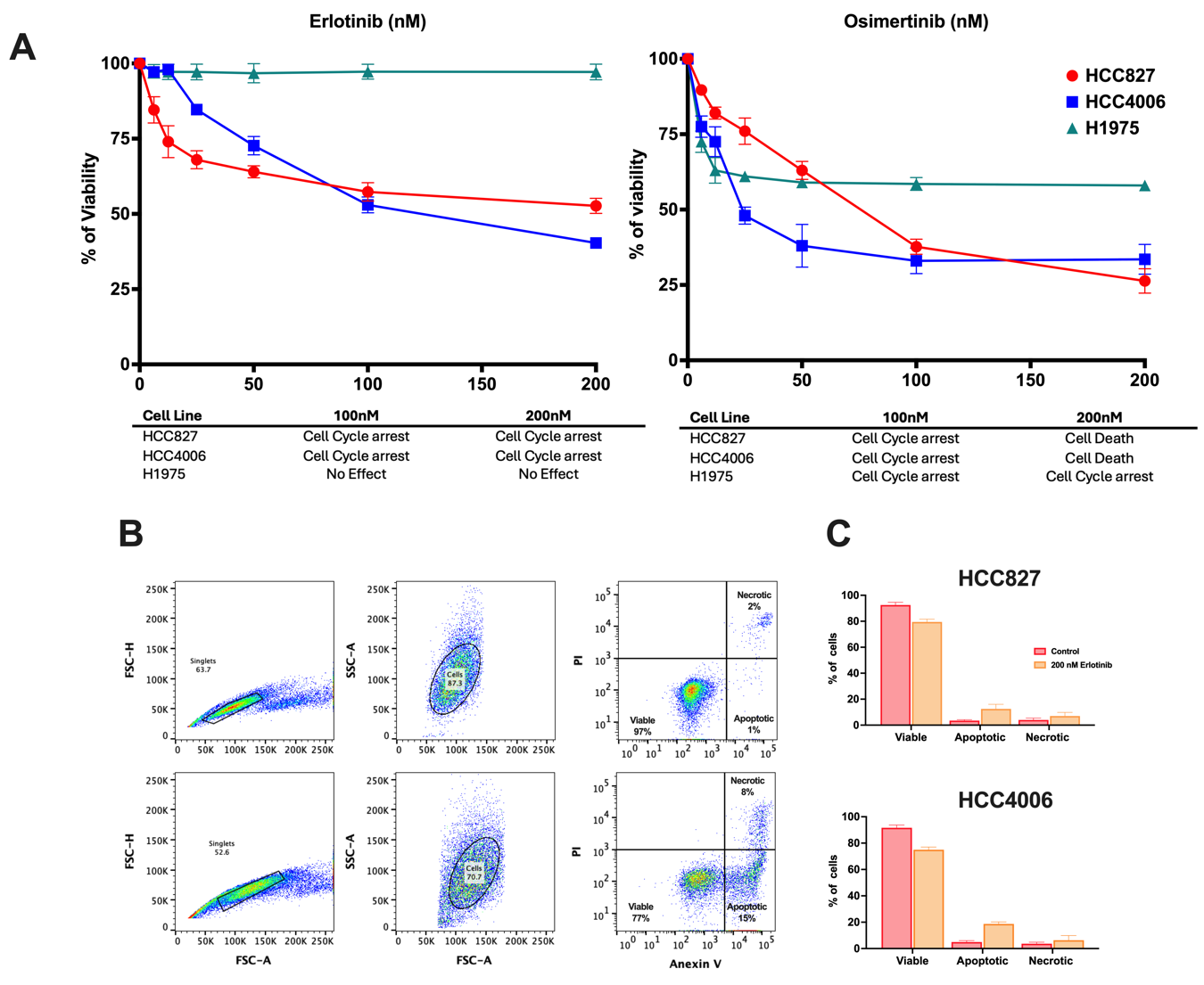


**Figure S1. Effect of TKIs on *EGFR*-mutated lung adenocarcinoma cell lines and cytometric analysis.** (A) Dose-response curves and biological effect induced by TKIs are shown. (B) Cytometric strategy for quantification of viable, apoptotic and necrotic cells. Percentage of cells in control culture (upper blots) compared with the effect induced by erlotinib (lower blots) in the cell line studied. (C) Percentages of viable, apoptotic and necrotic cells in HCC827 and HCC4006 cell lines. Three independent experiments were done. Mean ± SD is shown. *****p* < 0.0001.


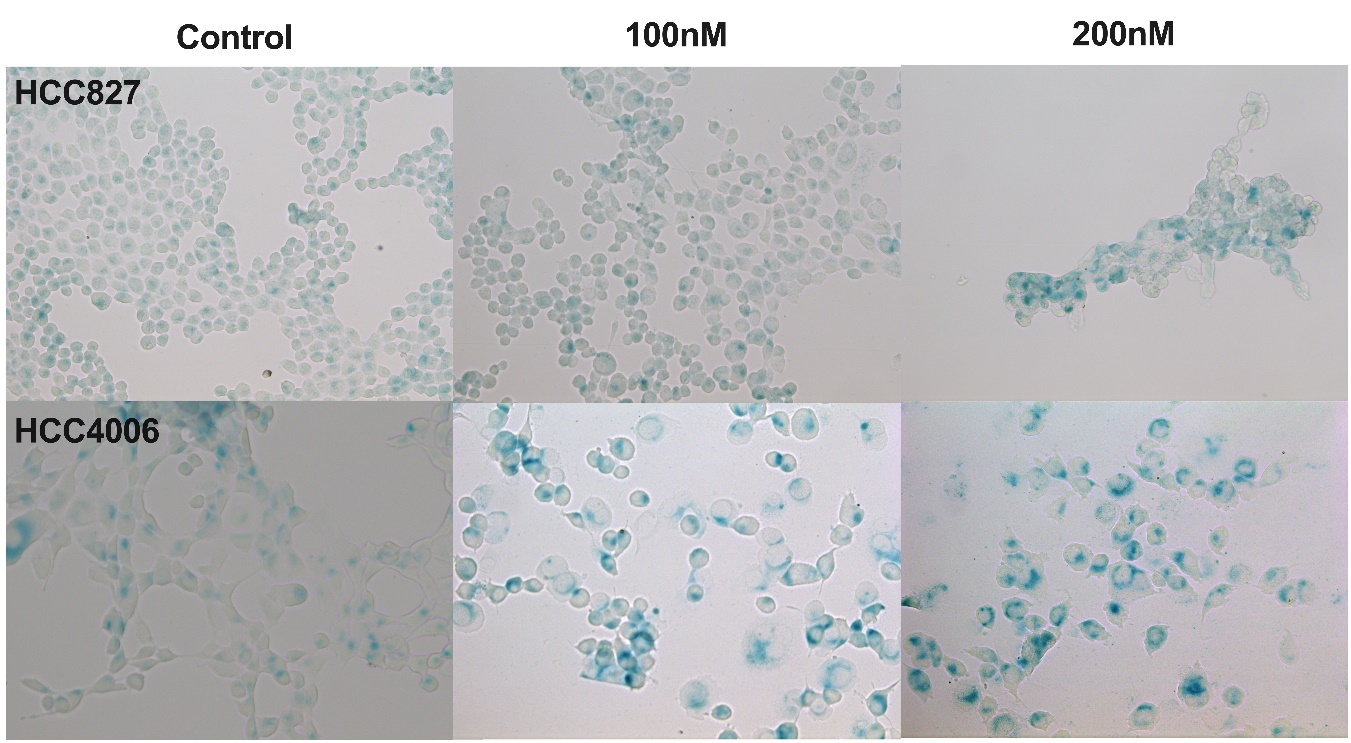


**Figure S2.** Detection of a senescent-like phenotype in DTP cells. After osimertinib exposure for 48 h, residual cells were cultured in fresh complete media for additional five days and the expression of beta-galactosidase positive cells were detected. Micrographs showing positive cells. Magnification 40x.


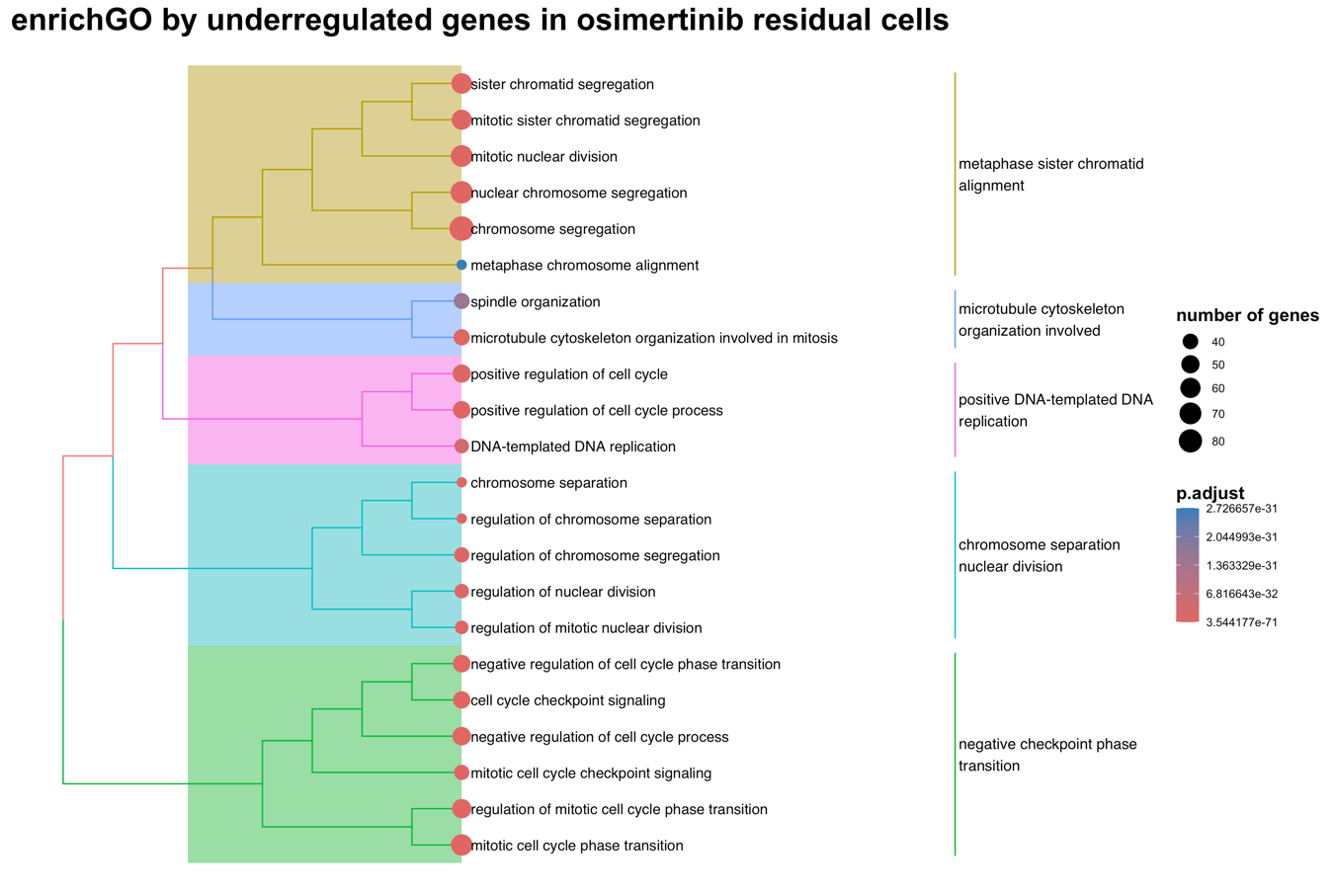


**Figure S3.** Functional annotation of the Osimertinib-induced gene underregulating in DTP cells. GO analysis reveal pathways associated with cell cycle regulation

**Table S1.** TaqMan® gene expression assays.

| **Gene** | **TaqMan assay ID** |
| --- | --- |
| *AGAP2-AS1* | Hs01096080_s1 |
| *MEG3* | Hs00292028_m1 |
| *NKILA* | Hs04937740_s1 |
| *CERS6-AS1* | Hs01372066_g1 |
| *LINC01133* | Hs04274447_m1 |
| *CD74* | Hs00269961_m1 |
| *NFYC-AS1* | Hs03670565_s1 |
| *FLJ31104* | Hs01377183_m1 |
| *SLC25A25-AS1* | Hs05052217_s1 |
| *ARIH2OS* | Hs03644906_s1 |
| *GAPDH* | Hs02786624_g1 |

**Table S2.** DsiRNA sequences employed for knockdown

| **Name** | **Sequence** | **Sense** |
| --- | --- | --- |
| *hs.Ri.AGAP2-AS1.13.1* | *5’ rGrUrCrCrArCrGrCrArGrArUrUrGrArArUrUrCrCrCrCrUTG 3’* | + |
|  | *5’ rCrArArGrGrGrGrArArUrUrCrArArUrCrUrGrCrGrUrGrGrArCrUrG 3’* | - |
|  |  |  |
| *hs.Ri.AGAP2-AS1.13.2* | *5’ rCrCrCrCrArCrUrUrGrUrUrArCrCrUrGrCrUrUrUrArUrAAA 3’* | + |
|  | *5’ rUrUrUrArUrArArArGrCrArGrGrUrArArCrArArGrUrGrGrGrGrArG 3’* | - |
|  |  |  |
| *hs.AGAP2-AS1.13.3* | *5’ rCrCrArCrUrUrGrUrUrArCrCrUrGrCrUrUrUrArUrArArATA 3’* | + |
|  | *5’ rUrArUrUrUrArUrArArArGrCrArGrGrUrArArCrArArGrUrGrGrGrG 3’* | - |
|  |  |  |
| *hs.Ri.LINC01133.13.1* | *5’ rArArArGrCrUrUrGrArCrUrGrArArGrGrUrArCrCrArArGGT 3’* | + |
|  | *5’ rArCrCrUrUrGrGrUrArCrCrUrUrCrArGrUrCrArArGrCrUrUrUrUrA 3’* | - |
|  |  |  |
| *hs.Ri.LINC01133.13.2* | *5’ rCrCrArArArGrUrCrCrArGrCrArUrGrGrUrArGrArCrArUCA 3’* | + |
|  | *5’ rUrGrArUrGrUrCrUrArCrCrArUrGrCrUrGrGrArCrUrUrUrGrGrArG 3’* | - |
|  |  |  |
| *hs.Ri.LINC01133.13.3* | *5’ GrCrArUrGrGrUrArGrArCrArUrCrArGrUrGrGrUrGrGrUAA 3’* | + |
|  | *5’ rUrUrArCrCrArCrCrArCrUrGrArUrGrUrCrUrArCrCrArUrGrCrUrG 3’* | *-* |
|  |  |  |
| *hs.Ri.CD74.13.1* | *5’ rArArUrArArArArGrGrUrArGrUrArArUrUrArGrArArCrAAA 3’* | *+* |
|  | *5’ rUrUFUrGUrUrCrUrArArUrUrArGrUrArCrCrUrUrUrUrArUrUrCrU 3’* | *-* |
|  |  |  |
| *hs.Ri.CD741.13.2* | *5’ rCrCrUrUrArUrCrUrCrCrArArCrArArUrGrArGrCrArArCTG 3’* | *+* |
|  | *5’ rCrArGrUrUrGrCrUrCrArUrUrGrUrUrGrGrArGrArUrArArGrGrUrC 3’* | *-* |
|  |  |  |
| *hs.Ri.CD74.13.3* | *5’ rCrArArGrUrArUrGrGrCrArArCrArUrGrArCrArGrArGrGAC 3’* | *+* |
|  | *5’ rGrUrCrCrUrCrUrGrUrCrArUrGrUUrGrCrCrArUrArCrUrUrGrGrU 3’* | *-* |

**Table S3.** LncRNAs overexpressed in erlotinib-DTP cells.

| **Ensembl ID** | **Gene name** |
| --- | --- |
| *ENSG00000214548* | *MEG3* |
| *ENSG00000231187* | *SYT15-AS1* |
| *ENSG00000231638* | *LUARIS* |
| *ENSG00000255737* | *AGAP2-AS1* |
| *ENSG00000260604* |  |
| *ENSG00000265666* | *RARA-AS1* |
| *ENSG00000278709* | *NKILA* |
| *ENSG00000280303* | *ERICD* |

**Table S4.** LncRNAs overexpressed in Osimertinib-DTP cells.

| **Ensembl ID** | **Gene name** |
| --- | --- |
| ENSG00000224032 | *EPB41L4A-AS1* |
| ENSG00000224259 | *LINC01133* |
| ENSG00000226200 | *SGMS1-AS1* |
| ENSG00000227617 | *CERS6-AS1* |
| ENSG00000253671 |  |
| ENSG00000254231 | *WWP1-AS1* |
| ENSG00000258634 |  |
| ENSG00000258701 | *LINC00638* |
| ENSG00000259976 |  |
| ENSG00000260604 |  |
| ENSG00000261799 |  |
| ENSG00000273033 | *LINC02035* |
| ENSG00000277496 | *SLCO4A1-AS2* |
